# Combination of ultrasound-peracetic acid washing and ultrasound-assisted aerosolized ascorbic acid: A novel rinsing-free disinfection method that improves the antibacterial and antioxidant activities in cherry tomato

**DOI:** 10.1016/j.ultsonch.2022.106001

**Published:** 2022-04-06

**Authors:** Jiayi Wang, Zhaoxia Wu, Hongbin Wang

**Affiliations:** a College of Food and Chemical Engineering, Shaoyang University, Shaoyang 422000, China; b College of Food Science, Shenyang Agricultural University, Shenyang 110000, China; c Shijiashike Co., Ltd, Liaoyang 111000, China

**Keywords:** Ultrasound, Fresh produce, Disinfection, Rinsing

## Abstract

Traditional ultrasound (US)-assisted disinfection is only effective during washing. Coating is an effective method to control microbial growth after washing; however, cross-contamination can occur during immersion in the coating aqueous solution. Tap water (TW) rinsing is generally used to remove sanitizer residues after US-assisted washing; however, the Food and Drug Administration stated that rinsing is unnecessary when the peracetic acid (PAA) concentration does not exceed 80 ppm. In this study, we proposed a novel US-assisted hurdle technology of 80 ppm PAA combined with low-frequency US (25 kHz) during washing, followed by US-assisted aerosolization processing (nonimmersion coating). Ascorbic acid (AA), a safe and low-cost agent, was selected as the aerosolization solution. Cherry tomatoes were selected as the model, and the proposed method was compared with traditional US-assisted disinfection methods (US-10 ppm free chlorine washing + TW rinsing and US-5 ppm chlorine dioxide washing + TW rinsing) to analyze the disinfection efficacy and quality changes. During storage, US-PAA + 1%AA facilitated additional 0.7–0.9, 0.6–0.8, 0.7–1.0, and 0.5–1.0 log CFU/g reductions in the counts of *Escherichia coli* O157:H7, *Salmonella* Typhimurium, aerobic mesophilic counts, and molds and yeasts, respectively, as compared with traditional US-assisted methods. Sensory properties, color index, total soluble solids, titratable acidity, and weight loss were not negatively affected by any of the treatments. Firmness was slightly reduced after all treatments; however, the firmness of the samples was maintained during storage, in contrast with the decreased firmness observed in the control. Phenolic content and antioxidant activity significantly increased after all treatments. Further analysis of two key enzymes (phenylalanine ammonia-lyase and 4-coumarate-CoA ligase) involved in phenolic synthesis showed that their levels significantly increased following all treatments, leading to an increase in phenolic content and antioxidant activity. This result also indicated that US-assisted washing could act as an abiotic elicitor to increase nutritional content. Overall, US-PAA + 1%AA treatment served as an effective method for disinfecting produce during washing and for controlling microbial growth after washing without prolonging the processing time, which is an advantage over traditional US-assisted washing.

## Highlights

• US-PAA + AA was more effective in controlling microbial growth than US-FC and US-CD.
• US-PAA + AA did not induce additional quality loss.
• US is a potential physical elicitor of phenolic content in fresh produce.

## 1 Introduction

Fresh produce is an important source of daily vitamins, minerals, and fiber. Due to an accelerated lifestyle, the demand for ready-to-eat produce is increasing. Since fresh produce is not thermally treated, consumption is accompanied by food safety hazards caused by foodborne pathogens. Among these pathogens, *Salmonella* is the most frequently detected, followed by *Escherichia coli* O157:H7 [1]. *Salmonella* and *E. coli* O157:H7 were the causative pathogens of foodborne diseases due to consumption of ready-to-eat fresh produce in the United States (47.65% and 30.87%, respectively) and the EU (47.62% and 8.33%, respectively) [2]. Recently, the Food and Drug Administration (FDA) reported 31 illnesses and 4 hospitalizations from June 10, 2021 to August 18, 2021 caused by consumption of pre-packaged salad contaminated with *S.* Typhimurium, in which the youngest infected person is less than one year old [3]. Meanwhile, baby spinach contaminated with *E. coli* OH157:H7 caused 15 illnesses and 4 hospitalizations from October 13, 2021 to November 8, 2021 [4]. Therefore, disinfection is an important step before distribution of ready-to-eat produce sales.

Ultrasound (US) is a non-thermal processing technology that can generate shear force and shock waves in an aqueous solution to detach and kill microorganisms on the surface of the produce [5]. However, a recent review concluded that the disinfection efficacy of US alone is limited [6]. Moreover, during washing, surface pathogens will enter the circulating wash water; if the pathogen in the water is not inactivated immediately, cross-contamination will occur. US has a limited cross-contamination prevention capacity. For example, US alone can reduce 1.41 log CFU/g *Salmonella* on iceberg lettuce, which is consistent with free chlorine (FC; 10 ppm) treatment; however, survival counts in washing water after US treatment were 5.70 log CFU/g, which is similar to that of tap water washing, in contrast to the undetectable counts after FC treatment [7]. Interestingly, the combination of US and FC (US + FC) showed lower the incidence of cross-contamination with *Pseudomonas fluorescens* in lettuce compared to US alone [8]. Wang et al. [9] utilized US to disinfect winter jujube and found that the cross-contamination with *Salmonella* and *E. coli* O157:H7 was consistent with that in tap water washing; however, cross-contamination was completely prevented after US + FC. Therefore, US should be combined with effective disinfectants in washing fresh produce. Among all disinfectants, peracetic acid (PAA), FC, and chlorine dioxide (CD) are commonly used owing to their low cost, excellent disinfection efficacy, and ability to prevent cross-contamination [10]. Improved disinfection efficacy was observed when US was combined with these three disinfectants. The combination of US and FC in washing kiwifruit reduced aerobic mesophilic counts (AMC) and molds and yeasts (M&Y) by 3.48 and 2.32 log CFU/g, respectively, which were significantly higher than those observed with US alone [11]. AMC and M&Y present on plum fruit were reduced by 3 and 2 log CFU/g, respectively, after treatment with the combination of US and CD (US + CD) [12]. Meanwhile, the combination of US and PAA showed an increased disinfection efficacy against AMC, M&Y, and *Salmonella* present on strawberries compared to US alone [13].

**Fig. 1:**
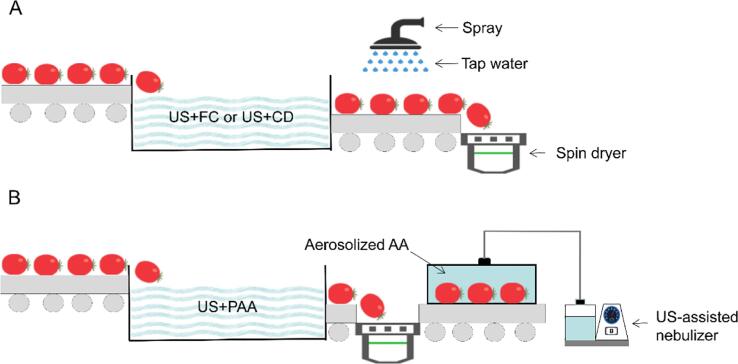
Schematic diagram of the cherry tomato disinfection process proposed in this study compared to the traditional US-assisted washing. (A) Traditional US-assisted process using US-FC or US-CD washing followed by tap water rinsing. (B) Proposed rinsing-free process using US-PAA washing with AA aerosolization processing. US, ultrasound; FC, free chlorine; CD, chlorine dioxide; PAA, peracetic acid; AA, ascorbic acid.

US-assisted disinfection was only effective during washing; thus, procedures to control microbial growth after washing should be developed. If a combination with this method extends the processing time as compared with the traditional method, the production efficiency will be reduced, thus reducing the acceptance by industry [1]. In general, a rinsing step using tap water (TW) is needed to rinse off the disinfectant residue after US-assisted washing (e.g., US-FC and US-CD; Fig. 1A). However, the FDA approved that rinsing was unnecessary as the PAA concentration did not exceed 80 ppm [10]. Therefore, a method replacing the rinsing step after US-PAA washing in maintaining microbial control can be a potential hurdle technology to the alternative traditional US-assisted washing followed by TW rinsing.

US-assisted nebulizers have been successfully used in indoor air disinfection and disease treatment. For fresh produce, aerosol droplets can adhere to the surface and continuously effective after treatment. Owing to the characteristics of ready-to-eat produce, the aqueous solution to produce aerosols should be safe, is low-cost, and do not affect sensory quality. Ascorbic acid (AA), a low-cost food additive, has been used for food preservation and against browning [14]. Moreover, among many ready-to-eat fruits, cherry tomatoes are available all year round and are consumed due to their good flavor and juiciness. In this study, US-PAA washing with US-assisted aerosolized AA processing (Fig. 1B) was used to disinfect cherry tomato, which was selected as a model, compared to the traditional US-assisted (US + FC and US-CD) washing followed by TW rinsing (Fig. 1A), and examined the disinfection efficacy and quality changes.

## 2 Materials and methods

### 2.1 Inoculation

Cherry tomatoes were purchased from a local market on the day of the experiment, and samples without rotting and apparent bruises were selected for the experiment. *S.* Typhimurium (ATCC14028) recommended by the FDA in food safety testing and non-toxic *E. coli* O157:H7 (NCTC12900) used for fresh produce disinfection experiments were selected in this study [15], [16], [17], [18]. The inoculation process was performed as described by Huang et al. [19], with some modifications. Briefly, a single colony of the two pathogens was inoculated in nutrient broth (Hopebio, Qingdao, China) and incubated for 12 h at 37 °C with shaking at 120 rpm. The bacterial suspension was washed three times using sterile 0.85% NaCl solution and resuspended in sterile distilled water to achieve ∼10^9^ CFU/mL cell concentration. Then, 10 cherry tomatoes and adjusted bacterial suspension were added into sterile stomacher bags at a ratio of 1:8 (w/v) and manually massaged for 15 min. The samples were transferred into a biosafety cabinet for air drying for 3 h. Finally, the inoculated samples were stored at 4 °C for 24 h to allow sufficient bacterial attachment.

### 2.2 Disinfection

#### 2.2.1 Washing water preparation

The washing water used for fresh-cut vegetables is recycled and the soluble matter from the produce can lead to a high chemical oxygen demand (COD) in the washing water, consuming oxidizing sanitizer. Thus, the use of produce homogenate in preparing the wash water with a certain COD value was recommended in previous studies [9], [20], [21]. Briefly, the cherry tomatoes were transferred to an analytical mill (A11 basic; IKA, Germany) and processed for 30 s. The resulting slurry was filtered under vacuum, and the supernatant was stored at −20 °C until analysis. The COD concentration in the wash water was adjusted to 756 ± 65 mg/L. The disinfectant concentration used in this study was 10, 5, and 80 ppm for FC (prepared using sodium hypochlorite; Sinopharm, Beijing, China), CD (HKM, Guangzhou, China), and PAA (Huanyu, Xianyang, China) [9], [10], [19], [20], [22], and was adjusted and stable at 12.5, 5.5, and 85 ppm, respectively, before washing. To maximize disinfection efficacy of FC, the pH of the wash water was adjusted to 5.5 using phosphoric acid. The concentrations of COD, FC, and CD were determined using test kits (Lohand, Hangzhou, China), and the concentration of PAA was determined using a strip (HKM).

#### 2.2.2 US-assisted washing followed by TW rinsing or US-assisted aerosolization processing

The processing periods for washing and rinsing were 5 and 1 min, respectively, based on previous studies [21], [23], [24], [25]. In the pre-experiment, the disinfection efficacy did not improve as the power exceeded 300 W; thus, a low frequency (25 kHz) and 300 W were used in this study. Since aerosolization was an alternative rinsing process, it was performed for 1 min without prolonging the processing time. The sensory flavor was negatively affected when the AA concentration exceeded 1%; thus, 0.5% and 1% AA were selected. In addition, in the pre-experiment, we confirmed that the disinfection efficacy of US-CD, US-FC, and US-PAA were significantly higher than that of US, CD, FC, and PAA; thus, these three combinations were used.

US-FC, US-CD, and US-PAA washing were performed as follows: 20 samples were placed into a stainless steel cage (18 cm × 15 cm × 5 cm) and into the US washer (SB-800DTS; Scientiz, Ningbo, China) containing 10 L of wash water. A submersible pump (3,500 L/h; Chuangning, China) was placed at the bottom of the washer to generate water flow.

The rinsing process was performed using a spray system (Sushen, Zhejiang, China) consisting of a bucket, a self-priming pump, and a spray nozzle. After US-FC and US-CD washing, the sample was placed on a shaker at 120 rpm (Jintan, Changzhou, China), and the spray nozzle was set at 70 cm above the sample. The pump was immersed in the bucket to initiate rinsing at a rate of 0.75 L/min [1]. The sample was then dewatered using a manual salad spinner sterilized using 75% ethanol. For the US-PAA washing, the samples were transferred into a chamber for aerosolization. The chamber (50 cm × 50 cm × 60 cm) was made of acrylic material, and the US-assisted nebulizer (aerosolization rate and US frequency: 3.6 mL/min (the maximum rate) and 1.7 MHz, respectively; 402AI, Yuwell, Shanghai, China) was connected to the top of the chamber. The size of the aerosolized droplet was approximately 3 µm, according to the manufacturer’s instructions. Before transferring the sample, the chamber was filled with aerosolized AA.

After the above treatments, the samples were transferred to a polyethylene terephthalate box and packaged using a polyvinyl chloride cling film (Nan Ya, Tai Wan, China) [16]. The samples were stored at 4 °C until analysis.

### 2.3 Microbiological analysis

Eight samples were randomly selected from the package, transferred to a sterile stomacher bag, diluted 1:9 (w/v) in sterile 0.85% NaCl solution, and homogenized in a stomacher for 2 min. Then, 1 mL of the diluted bacterial suspension was spread-plated on modified sorbitol MacConkey agar (Hopebio) and xylose lysine deoxycholate agar (Hopebio) and incubated for 24 h at 37 °C to analyze *E. coli* O157:H7 and *S.* Typhimurium, respectively. For naturally-present microbes, 1 mL of the bacterial suspension was pour-plated in plate count agar (Hopebio) and incubated at 37 °C for 2 d to obtain the AMC, and 1 mL was pour-plated in rose bengal agar (Hopebio) and incubated at 28 °C for 5 days to quantify the M&Y.

### 2.4 Quality and enzyme activity analysis

#### 2.4.1 wt loss

Weight loss in the sample was calculated as follows:

$$\mathrm{Weight}\;loss\;\left(\%\right)=1-\frac{\mathrm{Weight}\;after\;storage}{\mathrm{Initial}\;weight}$$

#### 2.4.2 Color index

The values of L*, a*, and b* were determined using a colorimeter (CR400; Konica Minolta, Osaka, Japan). Five samples were randomly selected from each package, and each sample was analyzed four times for a total of 20 readings per replicate. The color index was calculated using the formula described previously [26], as follows:

$$\mathrm{Color}\;index=\frac{2000\times a^{*}}{L^{*}\times \sqrt{a^{*2}+b^{*2}}}$$

#### 2.4.3 Firmness

Firmness was determined using TA. XT Plus Texture Analyzer (Stable Micro Systems, Godalming, UK) equipped with a cylindrical probe with a diameter of 3 mm. Five samples were randomly selected from each package, and the firmness of each sample was determined using the following parameters: pretest speed, 2 mm/s; test speed, 1 mm/s; post-test speed, 5 mm/s; auto trigger force, 5 g; and travel distance of the probe, 5 mm.

#### 2.4.4 Sensory analysis

Eight panels were invited to evaluate the sensory color, flavor, and firmness of the samples. A 3-point scale method was used for evaluation, in which 0 indicated very poor, 5 indicated acceptability threshold, and 10 indicated liking very much [27]. The samples were placed into white porcelain dishes with a mark at the bottom, and the dishes were reordered before evaluation. The sensory evaluation was conducted in a white-walled room with no windows, equipped with a 40 W incandescent lamp. During the evaluation, only one person was allowed to enter the room, and communication was not allowed after the evaluation. For flavor analysis, the panels were asked to gargle three times after tasting, and the next evaluation was performed after 30 s.

#### 2.4.5 Liquid nitrogen griding

Five samples were randomly selected from each package and rinsed for 1 min using TW to remove the AA present on the sample surface. After air-drying, the sample was soaked in liquid nitrogen for 30 s and then transferred to an IKA analytical mill for grinding. The resulting powder was used for analysis, as described in Sections 2.4.6–2.4.8.

#### 2.4.6 Total soluble solids (TSS) and titratable acidity (TA) analysis

The ground powder (0.5 g) was mixed with distilled water at a ratio of 1:5 and analyzed using a hand-held refractometer to determine the TSS content. TA analysis was performed according to GB/T 12293-1990.

#### 2.4.7 Polyphenolic content and antioxidant activity analysis

The ground powder (0.5 g) was mixed with 80% methanol at a ratio of 1:10 and then allowed to stand for 10 min. After centrifugation at 11,000 g for 10 min, the supernatant (50 μL) was mixed with 250 μL Folin reagent (Sinopharm) and 3 mL distilled water. After reaction for 6 mins, 750 μL of 20% sodium carbonate was added and incubated for 90 min in the dark. The absorbance was recorded at 765 nm, and the results were defined as gallic acid equivalents (mg/100 g) expressed on a fresh weight basis.

The 1,1-diphenyl-2-picrylhydrazyl (DPPH) method was used for the antioxidant analysis. Briefly, 24 mg DPPH was dissolved in 100 mL methanol to prepare a stock solution and stored at −20 °C until use. Before each measurement, a working solution with an absorbance of 1.1 ± 0.02 at 515 nm by mixing a 10 mL stock solution with 45 mL methanol. The supernatant (150 μL) was mixed with 2850 μL working solution and reacted for 8 h. The absorbance was recorded at 515 nm, and the results were defined as Trolox equivalent (μM/g) expressed on a fresh weight basis.

#### 2.4.8 Enzyme activity analysis

Phenylalanine ammonia-lyase (PAL) was analyzed following the protocol of Zheng et al. [28]. Briefly, ground powder (0.5 g) was homogenized in 2 mL 0.05 M boracic acid buffer (pH 8.8) containing 4% PVP, 2 mM EDTA, and 5 mM β-mercaptoethanol. After centrifugation at 11,000 g for 20 min at 4 °C, the supernatant (0.5 mL) was mixed with a reaction mixture (3 mL 50 mM boracic acid buffer, pH 8.8; and 0.5 mL 20 mM l-phenylalanine) and incubated at 37 °C for 1 h. The reaction was stopped by adding 0.2 mL 6 M HCl. The absorbance was determined at 290 nm, and PAL activity (U) was defined as the amount of enzyme that caused an increase in absorbance of 0.01 at 290 nm per hour.

The method reported by Liu et al. [29] was used for the 4-coumarate-CoA ligase (4CL) analysis. Briefly, 0.5 g of ground powder (0.5 g) was homogenized in 2 mL 0.2 M Tris-HCl buffer solution (pH 8.0) containing 25% glycerol (v/v) and 0.1 M DTT. After centrifugation at 11,000 g for 20 min under 4 °C, 0.5 mL supernatant was mixed with a reaction mixture (0.45 mL 15 μM MgCl_2_, 0.15 mL 50 mM ATP, 0.15 mL 1 mM CoA, and 0.15 mL 5 mM *p*-coumarate), and incubated at 40 °C for 10 min. The reaction was stopped by adding 0.1 mL 6 M HCl. The absorbance was determined at 333 nm, and 4CL activity (U) was defined as the amount of enzyme that caused an increase in absorbance of 0.1 at 333 nm per minute.

### 2.5 Statistical analysis

All data were analyzed using the SPSS v.20. Differences between the means of the groups were evaluated using one-way analysis of variance and post hoc Duncan’s multiple range test. Statistical significance was set at p < 0.05. Each experiment was independently performed three times, and the samples were analyzed on days 0, 3, and 5. Samples without any treatment were used as controls.

## 3 Results

### 3.1 Disinfection efficacy of different combinations

**Fig. 2:**
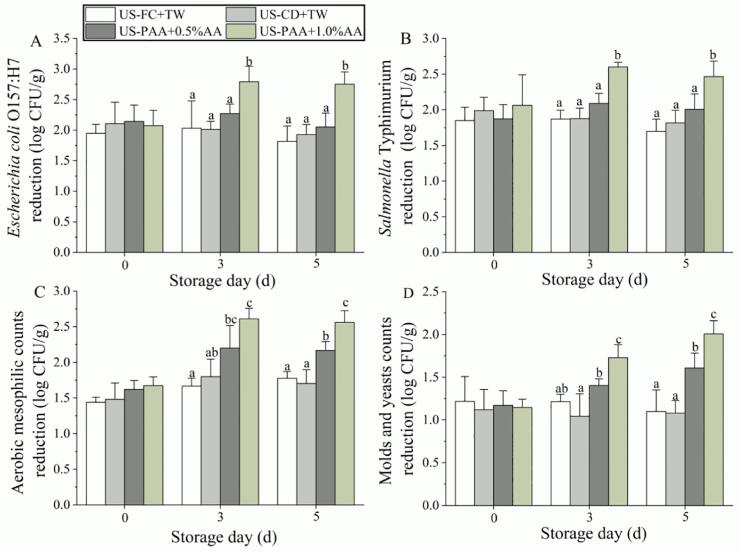
Microbial reduction caused by different combinations of washing treatments. (A) *E. coli* O157:H7. (B) *S.* Typhimurium. (C) Aerobic mesophilic counts. (D) Molds and yeasts. Count reduction indicates the difference in microbial counts between the control and treatment groups at the same time points. The different lowercase letters within the same group indicate significant differences (*P* < 0.05). US, ultrasound; TW, tap water; FC, free chlorine; CD, chlorine dioxide; PAA, peracetic acid; AA, ascorbic acid.

After treatment using traditional methods (US-FC + TW and US-CD + TW), *E. coli* O157:H7, *S.* Typhimurium, AMC, and M&Y were reduced by 1.95–2.11, 1.85–1.99, 1.44–1.48, and 1.12–1.22 log CFU/g, respectively (Fig. 2). Meanwhile, the proposed method (US-PAA + AA) did not lead to further microbial reduction compared to the traditional methods at day 0. During storage, US-PAA + 1% AA showed the highest reduction in pathogens, with 2.79 (day 3) and 2.75 (day 5) log CFU/g for *E. coli* O157:H7 and 2.60 (day 3) and 2.47 (day 5) log CFU/g for *S.* Typhimurium, which are significantly higher than those in traditional US-assisted washing method and US-PAA + 0.5%AA (Fig. 2A, B). Similarly, US-PAA + 1%AA showed the highest reduction in AMC and M&Y during storage (days 3–5). At day 5, US-PAA + 1%AA reduced M&Y to 2.01 log CFU/g, which was 1.83- and 1.86-fold of US-FC + TW and US-CD + TW, respectively (Fig. 1D). Moreover, reduction in AMC and M&Y caused by US-PAA + 0.5% AA was significantly higher than that of the traditional methods, whereas no significant difference was observed in Fig. 2A and B.

### 3.2 Effects of the different treatment combinations on the quality of cherry tomatoes

**Fig. 3:**
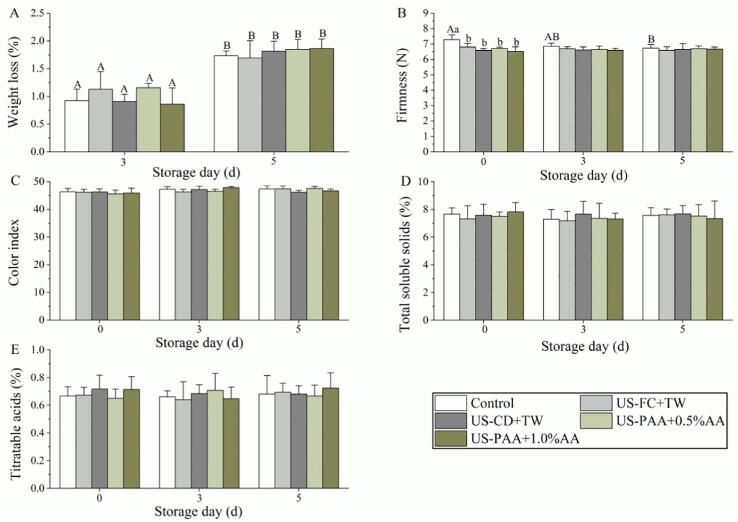
Effects of the different treatment combinations on the physicochemical properties of cherry tomato: (A) Weight loss, (B) Firmness, (C) Color index, (D) Total soluble solids, and (E) Titratable acids. Lowercase and uppercase letters above the column indicate significant differences between different treatments within the same day and between different days within same treatment, respectively. US, ultrasound; TW, tap water; FC, free chlorine; CD, chlorine dioxide; PAA, peracetic acid; AA, ascorbic acid.

The control group exhibited 0.92% weight loss at day 3, and the four combinations showed similar values ranging from 0.86 to 1.16%, which were not significantly different from the control group (Fig. 3A). After storage for 5 d, weight loss in the control group significantly improved to 1.73%, and all treatment groups were not significantly higher than the control group. Firmness analysis showed firmness values of 6.53–6.80 N during the US-assisted washing (i.e., all treatment groups), which were significantly lower than the control (7.28 N) (Fig. 3B). During storage, the firmness of the control group showed a decreasing trend, reaching 6.74 N on day 5, which was significantly lower than the value at day 0; however, firmness of the treatment groups did not decrease during storage. In addition, the color index of the control group was 46.33 on day 0 and did not change during subsequent storage, and the treatment group was similar to the control group throughout the five-day period (Fig. 3C). TSS and TA of the control group were 7.65 and 0.67 on day 0, respectively, and these two indicators were not significantly changed after treatment (Fig. 3D, E). During days 3–5, TSS and TA did not change in the control group, and no significant difference was observed between the treatment and the control groups.

**Fig. 4:**
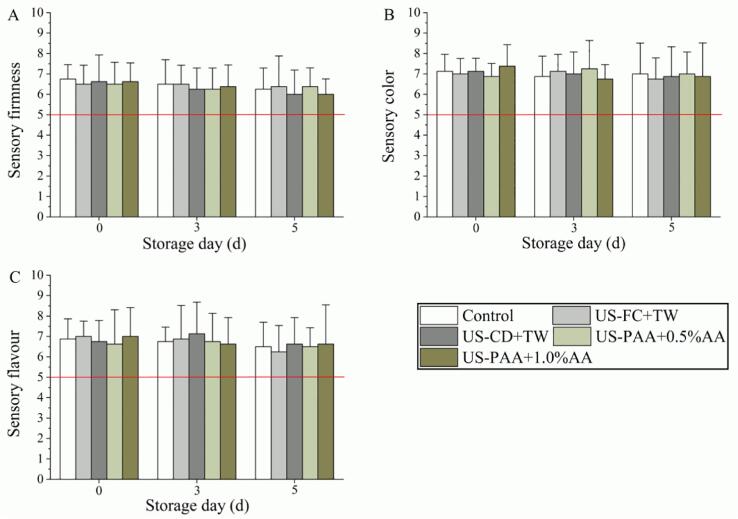
Effects of different treatment combinations on the sensory quality of cherry tomato. (A) Sensory firmness, (B) Sensory color, and (C) Sensory flavour. No significant differences were observed between different treatments within the same day and between different days within same treatment. US, ultrasound; TW, tap water; FC, free chlorine; CD, chlorine dioxide; PAA, peracetic acid; AA, ascorbic acid.

Sensory quality (sensory firmness, color, and flavor) was analyzed during the five-day period, and the results are shown in Fig. 4. At day 0, firmness scores in the treatment and control groups exceeded the acceptability threshold (i.e., 5 points), and the values observed in the treatment groups were similar to those in the control group (Fig. 4A), which was inconsistent with the results observed in Fig. 3B. The firmness score did not significantly change during storage in all groups, and no significant difference was observed between the control and treatment groups. The sensory color score in the control group exceeded 5 points from days 0–5, and the score was not significantly changed after treatment (Fig. 4B), which is consistent with the results observed in Fig. 3C. Furthermore, US-PAA + AA did not promote flavor loss from days 0–5 (Fig. 4C).

### 3.3 Changes in the antioxidant and enzyme activities after treatment with different combinations

**Fig. 5:**
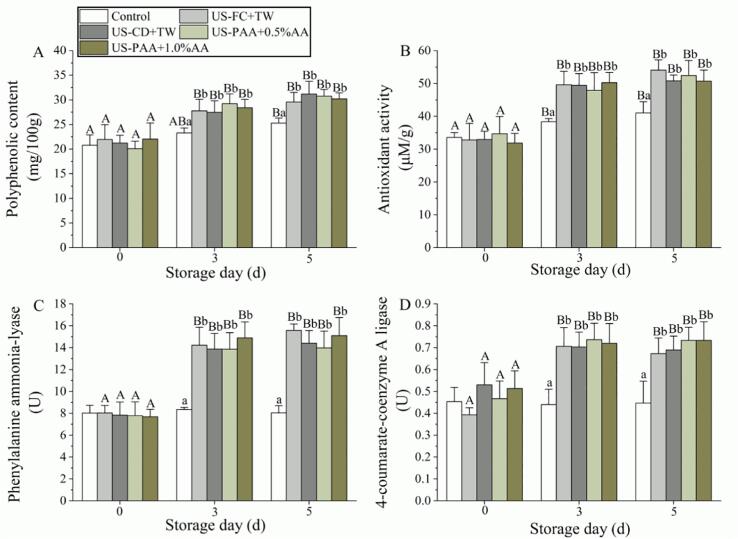
Effects of different treatment combinations on the antioxidant and phenolic metabolism enzyme activities of cherry tomato. (A) Polyphenolic content, (B) Antioxidant activity, (C) Phenylalanine ammonia-lyase, and (D) 4-coumarate-CoA ligase. Lowercase and uppercase letters above the column indicate significant differences between different treatments within the same day and between different days within same treatment, respectively. US, ultrasound; TW, tap water; FC, free chlorine; CD, chlorine dioxide; PAA, peracetic acid; AA, ascorbic acid.

An increasing trend of polyphenolic content in the control group was observed from days 0–5. At day 5, the polyphenolic content was significantly improved from 20.79 (day 0) to 25.29 mg/100 g (Fig. 5A). For the treatment groups, the polyphenolic content was similar with that of the control group at day 0; however, at day 3, it was significantly improved to 28.41–27.79 mg/100 g, which were significantly higher than that of the control group. At day 5, the polyphenolic content did not significantly improved but were still significantly higher than that of the control group. Meanwhile, the antioxidant activity did not change after treatment on day 0; however, it was significantly improved after storage for 3 d and was not further increased from days 3–5 (Fig. 5B). Consistent with the results observed in Fig. 5A, the antioxidant activity in the treatment groups was significantly higher than that in the control group from days 3–5. In the control group, the enzyme activity of PAL and 4CL were 8.03 U and 0.45 U at day 0, respectively, and their activities did not change throughout the storage period. The treatment groups did not exhibit significant difference with the control group at day 0. However, the enzyme activities significantly improved after storage for 3 d and was significantly higher than that of the control. After storage for 5 d, PAL and 4CL activities in the treatment groups did not change further but was still higher than that of the control group, which was consistent with the polyphenolic and antioxidant analysis results. When comparing the proposed methods (US-PAA + AA) with the traditional methods (US-FC + TW and US-CD + TW), no significant differences in PAL, 4CL, phenolic content, and antioxidant activities were observed from days 3–5.

## 4 Discussion

FC damages the cell membrane, leading to intracellular component leakage [16], [27]. As a physical treatment method, US can induce the generation of cavitation bubbles; a transient high pressure is formed during bubble rupture, called shear force and shock wave, and this pressure disrupts the cell membrane [30]. Therefore, the combination of US and FC accelerates cell membrane damage. Guo et al. [31] found that US combined with FC can cause more severe membrane damage and protein conformation changes in *E. coli* than either of the individual treatments. Furthermore, a recent study indicated that the antibacterial mechanism of CD, another chlorine-based disinfectant, does not primarily involve the cell membrane and instead harms cells by damaging intracellular components, a mechanism that is significantly different from that of FC [32]. Therefore, the US + CD treatment may kill bacteria by inducing intracellular and cell membrane damage. In this study, we found that the disinfection efficacies of US-FC and US-CD were comparable.

Similar to FC, PAA damages the cell membrane, and the combination of US and PAA accelerates membrane damage [33]. The antibacterial mechanism of acid is as follows: after penetrating the cell, the high intracellular pH environment stimulates molecular dissociation, and the dissociated molecules accumulated in the cell can attack DNA and RNA, inhibit energy metabolism, induce protein denaturation, block ion channels, and cause cell deformation [34], [35]. Therefore, AA causes intracellular damage, and the US-FC + AA treatment may kill bacteria by inducing intracellular and cell membrane damage.

In general, hurdle technology combines different disinfection methods with different antibacterial mechanisms to further reduce microbial contamination [36]. In this study, compared with the results obtained for US-FC and US-CD, further reductions were not observed after combining US-PAA with AA, owing to the weak antibacterial activity of AA. Hyun et al. [37] also found that 1% AA was ineffective at disinfecting tomatoes against *E. coli* O157:H7. However, AA has been shown to have strong antibacterial activity during food storage. Recently, AA was successfully employed to disinfect Salmonella in soft cheese during storage [38]. At the end of storage, *E. coli* O157:H7 present on AA-coated tomatoes was undetectable, whereas samples coated with carvacrol, citric acid, curcumin, and riboflavin showed 2.01, 1.46, 2.60, and 2.65 log CFU/g reductions in *E. coli* O157:H7 counts, respectively [37]. In this study, we also observed greater reductions in microbial counts following the US-PAA + AA treatment from days 3–5 compared with the results of the US-FC and US-CD treatments.

Organic acids have a higher disinfection efficacy against M&Y than against AMC [39]. Wang et al. [40] compared the disinfection efficacy of seven organic acids against M&Y and AMC on lettuce and found that organic acids had higher antibacterial activity against M&Y than against AMC. Similarly, at the end of storage, we observed that treatment with US-PAA + 1.0% AA led to a greater reduction in the M&Y count compared with the reduction in the AMC. A previous study showed that the counts of *Listeria monocytogenes* on lettuce treated with 0.5% citric acid, propionic acid, and acetic acid were even higher than those of the control at the end of storage and that *L. monocytogenes* was significantly inactivated during storage when the concentration was increased to 1% [41]. The authors concluded that this was mainly because 0.5% organic acids exhibit stronger inhibitory effects against AMC and M&Y than against *L. monocytogenes*, leading to an imbalance in the microbial composition and resulting in the rapid growth of *L. monocytogenes*. However, the organic acid concentration of 1% exceeds the upper limit of *L. monocytogenes*. This could also explain why US-PAA + 0.5% AA treatment did not lead to a greater reduction in *E. coli* O157:H7 and *S.* Typhimurium counts during storage compared with the US-FC and US-CD treatments. A significantly greater reduction was observed as the AA concentration increased to 1%.

Changes in the sensory quality following acid treatment should also be considered. Vijayakumar and Wolfhall [42] used white vinegar to process lettuce, and its sensory taste, texture, and overall acceptance scores were significantly lower than those of the sample treated with lemon juice and bleaching powder. Wang et al. [40] found that propionic acid leads to strong sensory flavor loss compared with the control. In this study, aerosolized AA did not affect the sensory color or flavor of cherry tomatoes, which may be because of the thicker waxy layer of their epidermis and their sourer taste compared with those of other produce. Firmness is also an important quality attribute of cherry tomatoes, and softening reduces quality and limits commercialization. Mustapha et al. [23] demonstrated that low-frequency US caused firmness loss in cherry tomatoes. In this study, we also observed slight firmness loss after US treatment owing to the cavitation effect of US during treatment; this may have caused injury to the tissue because of loss of cell wall stability [43]. US can be used to maintain the firmness of fresh produce during storage. For example, in one previous study, the firmness of strawberries at the end of storage was found to be 2.35 kg/cm, which was significantly higher than that of the control [44]. After treatment with US, the firmness of plum fruit was approximately 1.5-fold that of the control during storage [45]. Similar results were also observed in this study, and cherry tomato firmness was not lost during storage, in contrast with the decreasing trend observed in the control.

When plants are subjected to external stimuli, particularly abiotic stresses, the phenylpropanoid metabolic pathway is activated by accelerating secondary metabolite synthesis, mainly that of phenolic compounds [29]. PAL and 4CL are two key enzymes involved in the phenylpropanoid pathway. Chemical methods are commonly performed to prolong the shelf life and improve the quality of fresh produce [47], [48]. However, no reports have shown that AA can serve as an abiotic stimulant in plants to synthesize secondary metabolites. Among the physical methods, US has been shown to increase the phenolic contents of cherry tomatoes [24], [49]. In this study, we also observed that the phenolic contents of cherry tomatoes increased during storage and were significantly higher than those of the control, likely owing to the upregulation of PAL and 4CL. Similar results were reported by Lu et al. [24], who found that PAL activity in tomatoes treated with US was 30.57% higher than that of the control. The antioxidant activity of the tomatoes was correlated with their phenolic contents, and increased antioxidant activity was observed after US treatment, consistent with previous reports [23], [24], [50].

## 5 Conclusion

In this study, PAA was combined with US during the washing stage, and a US-assisted AA aerosolization approach was used to achieve microbial control during storage. There were three main findings of this study. First, compared with the traditional US-assisted disinfection method (US-FC + TW and US-CD + TW), the proposed method (US-PAA + 1%AA) did not lead to further microbial reduction (in terms of *E. coli* O157:H7, *S.* Typhimurium, AMC, and M&Y) on day 0; however, a significantly higher reduction was observed during storage. Second, US-PAA + 1%AA treatment did not lead to additional quality loss as compared with the control. Third, US treatment induced the upregulation of PAL and 4CL, leading to an increase in the polyphenolic content and antioxidant activity of cherry tomatoes.

Although AA did not affect the sensory flavor of cherry tomatoes, its effects on sweet fruits should be explored in the future. Natural products are emerging as green and safe antibacterial treatments, and the combination of US-PAA with an aerosolized mixture containing AA and natural products should be evaluated in future studies. In addition, the mechanisms underlying the enhancement of phenolic contents have not been elucidated at the molecular level; therefore, such molecular analyses using multi-omics techniques should be undertaken in future studies.

### CRediT authorship contribution statement

**Jiayi Wang:** Conceptualization, Supervision, Funding acquisition, Writing – original draft, Writing – review & editing. **Zhaoxia Wu:** Data curation, Writing – original draft, Writing – review & editing. **Hongbin Wang:** Data curation.

## Declaration of Competing Interest

The authors declare that they have no known competing financial interests or personal relationships that could have appeared to influence the work reported in this paper.
